# Nonlinearity Measurements of High-Power Laser Detectors at NIST

**DOI:** 10.6028/jres.109.030

**Published:** 2004-08-01

**Authors:** Xiaoyu Li, Thomas Scott, Shao Yang, Chris Cromer, Marla Dowell

**Affiliations:** National Institute of Standards and Technology, Boulder, CO 80305

**Keywords:** attenuation method, calibration, calorimeter, chopper wheel, high-power laser, nonlinearity measurement, thermal detector

## Abstract

We briefly explain the fundamentals of detector nonlinearity applicable to both electrical and optical nonlinearity measurements. We specifically discuss the attenuation method for optical nonlinearity measurement that the NIST system is based upon, and we review the possible sources of nonlinearity inherent to thermal detectors used with high-power lasers. We also describe, in detail, the NIST nonlinearity measurement system, in which detector responsivity can be measured at wavelengths of 1.06 µm and 10.6 µm, over a power range from 1 W to 1000 W. We present the data processing method used and show measurement results depicting both positive and negative nonlinear behavior. The expanded uncertainty of a typical NIST high-power laser detector calibration including nonlinearity characterization is about 1.3 %.

## 1. Introduction

For many years, thermal detectors have been used extensively to measure the output of high-power lasers [1]. One of the most important properties of these detectors is their optical response nonlinearity, i.e., the deviation from an expected linear relationship between the detector’s response and the incident optical power. Although some thermal detectors have embedded electrical heaters that can be used to characterize their nonlinearity, for the majority of detectors the non-linearity must be characterized with optical methods.

In the NIST high-power, continuous-wave (CW) laser detector calibration laboratory, K-series calorimeters are used as primary standards for the absolute calibration of laser power and energy meters spanning a laser power range of 5 W to 1000 W with a cumulative energy input of 0.3 kJ to 3 kJ [2]. Since the calibration of high-power laser detectors is costly and time-consuming, calibrations at multiple power levels for a single detector are not usually desirable. If a detector is linear, i.e., its response is directly proportional to the incident optical power, then a calibration needs to be performed only at one power level within that specified range. Unfortunately, most thermal detectors behave linearly within a rather limited power range.

We have developed a system [3] for measuring the nonlinearity of the detectors over the 1 W to multikilowatt power range. The system is based on an attenuation method using a beamsplitter, a reflective chopper wheel, and a translation stage to obtain a quantitative measure of detector output as a function of optical power input. Since a rotating chopper wheel is involved, this system is only suitable for measuring relatively slow detectors, such as thermal detectors, and using CW lasers or pulsed lasers with high pulse-repetition frequencies. By knowing the calibration factor at a single power level as well as the non-linearity behavior over a desired power range, the user can then obtain a calibration factor for any desired power level within this range. Consequently, this combination of NIST services is the most cost-effective calibration process for those customers who need detector calibration at more than one power level.

## 2. Detector Nonlinearity

### 2.1 Fundamentals of Detector Nonlinearity

The definition of detector nonlinearity [4] can be expressed in Eq. (1)

$$\Delta_{\text{NL}}\left(V;V_{r}\right)=\frac{R\left(V\right)-R\left(V_{r}\right)}{R\left(V_{r}\right)},$$ (1)

where

*R*(*V*) = *V*/*P* = the responsivity of the detector at an output of *V*, which is the voltage or reading of the power meter, at the input power or energy of *P*,
*R*(*V*_r_) = the responsivity at a reference point *V*_r_.

If a calibration is performed at *V*_c_ after the nonlinearity *∆*_NL_(*V*;*V*_r_) is measured with reference point *V*_r_ different from *V*_c_, the measured nonlinearity can be converted to *∆*_NL_(*V*;*V*_c_) by Eq. (2)

$$\Delta_{\text{NL}}\left(V;V_{c}\right)=\frac{\Delta_{\text{NL}}\left(V;V_{r}\right)-\Delta_{\text{NL}}\left(V_{c};V_{r}\right)}{1+\Delta_{\text{NL}}\left(V_{c};V_{r}\right)},$$ (2)

Equation (3) shows a calibration factor determined by detector calibration procedure

$$F=V_{c}/P_{c},$$ (3)

where

Pc = the power or energy of calibration,
Vc = the corresponding output of the detector.

If the detector is linear, *F* can be used for any output *V* to determine the input power or energy *P* = *V*/*F*. For a nonlinear detector, a correction factor *CF*(*V*) is applied to the calibration factor *F* and we have a more general expression in Eq. (4)

$$P=\frac{V}{F\times CF\left(V\right)},$$ (4)

When the nonlinearity is small, the most commonly used function for the *P-V* relation is a polynomial of the form in Eq. (5)

$$P\left(V\right)=\sum_{k=1}^{n}a_{k}V^{k}=a_{1}(V+\sum_{k=2}^{n}b_{k}V^{k}),$$ (5)

where *b_k_* = *a_k_*/*a*_1_.

*a*_1_ *V* is the linear term and the rest are nonlinear terms. It is assumed here that the background reading is adjusted to zero. We can further simplify the polynomial expression by dividing *P*(*V*) by *a*_1_ without altering the nonlinearity in Eq. (6)

$$P\left(V\right)=\frac{P\left(V\right)}{a_{1}}=(V+\sum_{k=2}^{n}b_{k}V^{k}),$$ (6)

where

*p*(*V*) = the normalized polynomial,
*a*_1_ = the calibration coefficient.

There are four commonly used measurement methods of nonlinearity: (1) superposition, (2) attenuation, (3) correlation, and (4) AC-DC (or differential) [4]. Our system is based on the attenuation method and the following discussion will focus on this method.

During the measurement process, the output voltages of the detector *V* (without attenuation) and *V*_τ_ (with attenuation) are recorded. Assuming the attenuation *τ* of the attenuator is known and that we have a normalized polynomial relationship between *p* and V, then Eq. (7) shows the *i*th measurement

$$\{\begin{array}{c} p_{i}=V_{i}+\sum_{k=2}^{n}b_{i}V_{i}^{k}\\ \tau p_{i}=V_{\tau i}+\sum_{k=2}^{n}b_{k}V_{\tau i}^{k} \end{array},$$ (7)

Eliminating the unknown *p_i_*, we obtain Eq. (8)

$$\left(V_{\tau i}-\tau V_{i}\right)+\sum_{k=2}^{n}b_{k}\left(V_{\tau i}^{k}-\tau V_{i}^{k}\right)=0,$$ (8)

By adjusting the source power, we can take sufficient measurements, such that the coefficients of *b_k_* can be obtained from the data by performing a linear least square fitting.

### 2.2 Possible Sources of the Thermal Detector Nonlinearity

The majority of thermal detectors used for high-power CW laser measurements consist primarily of thin metal discs with appropriate housings. The disk surface (facing the incident laser beam) is typically coated with highly absorbent material. A temperature sensor (thermopile or other temperature sensing device) is attached to the other side of the disc. The housing can be constructed in one of three different ways: (1) heat sink with ambient-air convection cooling, (2) heat sink with forced air (electrical fan) cooling, or (3) water jacket cooling [5]. When the laser radiation strikes the detector’s surface, there is direct energy loss caused by reflectance (specular and diffuse) at the detector’s surface and this loss is usually in the range of 5 % to 25 % of the incident radiation. Although the amount of optical reflectance of the detector surface varies with incident laser wavelength, ideally it should be constant over the entire power measuring range at a given laser wavelength. The radiation absorbed by the detector’s surface coating causes a temperature rise of the sensor disc and then thermal sensors produce an electrical output that is proportional to this temperature change. The resulting magnitude and distribution of laser-generated thermal energy can directly affect the detector’s non-linearity properties.

The three major sources of detector nonlinearity are (1) nonlinear behavior of the temperature sensor as a function of temperature, (2) radiation loss from the absorbing disc as the localized temperature varies in response to different incident power levels, and (3) temperature rise over time of the detector housing due to insufficient cooling (this phenomenon is especially noticeable when the detector is exposed to laser radiation over a long time period). Among the three possible detector nonlinearity sources listed above, nonlinearity sources (1) and (2) are the predominant sources for most detectors. The nonlinearity source (3) is a function of the exposure time, and for short exposure times (customary operating condition for most detectors) this nonlinearity contribution will be quite small.

## 3. Nonlinearity Measurement System

**Fig. 1:**
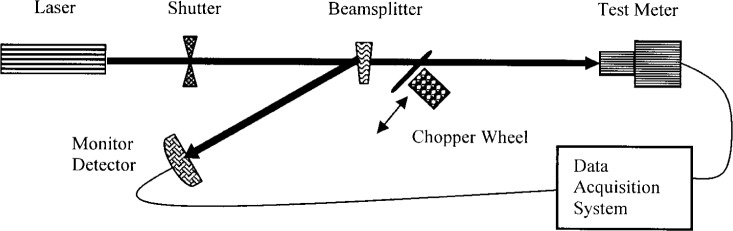
Detector nonlinearity measurement system.

Figure 1 is a schematic drawing of the NIST nonlinearity measurement system for characterizing high-power laser detectors. The laser beam passes through a beamsplitter oriented so that the first reflected beam strikes a monitor detector and the primary beam is transmitted to the test meter. A rotating optical chopper wheel located on a translation stage between the beam-splitter and the test detector is moved in and out of the laser beam. Because of its location, the monitor detector’s reading is only affected by the source power levels, but not by the chopper wheel movement. The following paragraphs give more details of the three key optical elements used in our system.

### 3.1 Laser

A laser source (producing radiation at the desired wavelength) with adjustable output power and shutter is chosen to provide the required power range for the nonlinearity measurement. The power is varied by either adjusting the laser power itself (if the laser output power can be stabilized at all power levels) or by using a variable high-power laser attenuator. Ideally the output power stability should vary less than ± 0.5 % in any 15 min time period as laser power fluctuations directly affect the measurement uncertainty. An internal or external shutter is used to turn the laser beam on and off. Currently, our laboratory system employs a 1 kW CO_2_ cw laser and a 500 W Nd:YAG cw laser. In addition, off-site laser sources can be used in conjunction with a portable beamsplitter/attenuator system designed and built by NIST to perform these measurements.

### 3.2 Beamsplitter

A beamsplitter and a monitor detector are used to detect laser power fluctuations during the measurement. The beamsplitter ratio (i.e., the ratio of transmitted power to reflected power) does not have to be known or even be constant over the entire power range of the nonlinearity measurement. However, the ratio does need to be thermally stable (with time) at each fixed power level used. To minimize measurement uncertainty, the detector used as a monitor should have good spatial uniformity, proper optical aperture size, and appropriate sensitivity. Because of the way it is used, the monitor detector does not have to be linear over the entire measurement power range, but it must be stable over the exposure time at each fixed power level. The time constant of the monitor detector should be as close to the test detector’s time constant as possible in order to accurately compensate for the fluctuation of the laser output.

### 3.3 Attenuator

A rotating optical chopper wheel mounted on a moveable translation stage provides the consistent attenuation required in this measurement technique. The chopper wheel is essentially a thick aluminum disc that has four open sectors precisely cut by EDM (Electrical Discharge Machine). 45° beveled edges were machined on the back surface along all the openings, but 0.5 mm straight edges from the front surface were kept. The disc surface was first black anodized and then the front surface was diamond-turned to produce a highly reflective surface to minimize the amount of absorbed radiation. The transmitted beam, which travels through the open sectors of the chopper wheel, is incident on the test detector and the reflected beam is incident onto a beam dump. The chopper wheel, thus, acts as an attenuator for radiation going to the test detector. The attenuation *τ* (Eq. (7), (8)) of the chopper wheel is the ratio of the power in the attenuated beam to the total laser power, or the duty cycle of the chopper. Since *τ* is a crucial parameter in the attenuation method, we have used both mechanical and optical methods to determine its value. The mechanical method uses a precision optical comparator to determine the total angle of the radius opening, and the optical method uses the NIST C-series calibration system [6]. The results from the two methods agreed within 0.1 %. The value of the in our system is about 70 %.

## 4. Measurement Procedures and Analysis

The nonlinearity system uses a computer controlled data acquisition system to acquire and process the data. Data acquisition instruments collect the output voltage of the monitor detector and the test meter simultaneously. The computer software written for this system controls the translation stage and data acquisition system.

For each nonlinearity measurement, we typically divide the desired measurement range into six equally spaced power points. The laser source is adjusted to produce each of the power points stepping from low to high. At each power point we take five sets of data with two measurements in each set of data. The first measurement is taken when the chopper wheel is out of the laser beam, and the second measurement is taken when the chopper wheel is inserted into the laser beam. The background readings of the test meter and monitor detector are always taken before the laser shutter is opened. There are two different methods for taking the detector’s background readings. For a water or fan cooled test meter, in which the detector’s housing temperature is stable, we take the background reading only once for each power level, so the laser shutter is opened and closed only once during the five repeated measurements. For a convection-cooled test meter, the housing temperature will typically rise during a long-term exposure to a laser power, so we take background readings before each individual measurement. Consequently, the laser shutter is opened and closed ten times for each power level.

As stated earlier, the thermal detector nonlinearity is affected primarily by the magnitude and distribution of laser generated thermal energy. In order to cover the desired power range of 1 W to 1000 W, we perform the nonlinearity measurements using a Nd:YAG laser at 1.06 µm and a CO_2_ laser at 10.6 µm. The Nd:YAG laser produces a stable output from 1 W to 300 W whereas the CO_2_ laser produces a stable output at powers of 300 W to 1000 W. Our measurement service for the detector nonlinearity, thus, covers the range from 1 W to 1000 W by using the two different lasers. Most high power thermal detectors work at these two wavelengths.

The absolute calibration [7] of the test meter at the desired laser wavelength and power level *P*_c_ [used in Eq. (3)] can be done prior to or after the nonlinearity measurement, since they are two independent measurements. The absolute calibration determines the detector responsivity *F* defined in Eq. (3). If the test detector output is in units of mV and the input power in units of W, then the responsivity will have units of mV/W.

The data from the detector nonlinearity measurements consist of pairs of measurements in which the detector output is sampled with and without the chopper wheel in the beam. After taking several sets of these measurement pairs, the power level is changed and the process is repeated. The first step of the data processing is to correct the test meter’s readings by the monitor detector’s readings to eliminate the effect of laser instability during the measurement. Since the attenuation *τ* of the chopper wheel is a known value, the amount of experimental deviation from that value is a measure of the test meter’s nonlinearity. In other words, for a perfectly linear detector, measured attenuation of the chopper wheel using the test meter’s voltage outputs at different power levels should all equal *τ*. In practice, very small discrepancies in measured attenuations of the chopper wheel are expected due to system noise, but these discrepancies should be randomly distributed about *τ* when the detector response is linear.

The detector responsivity as a function of laser power is determined from a least-squares fit to the measurement data assuming a polynomial expression for the relationship between detector output and incident laser power. The absolute calibration result is used as the reference point for the measurement data. The curve fitting procedure requires that the difference of the attenuation calculated from the fitted curve and the actual *τ* be minimized. An optimum order of the polynomial curve is selected considering the differences with respect to the noise.

**Fig. 2:**
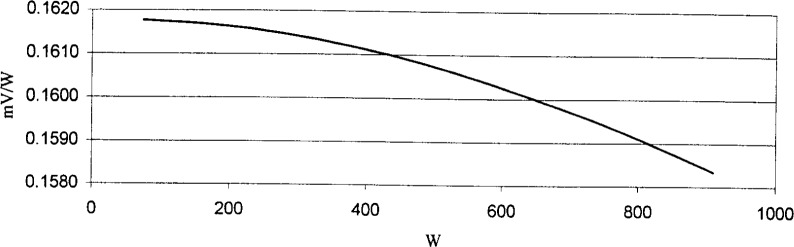
Negative nonlinear behavior of the detector.

**Fig. 3:**
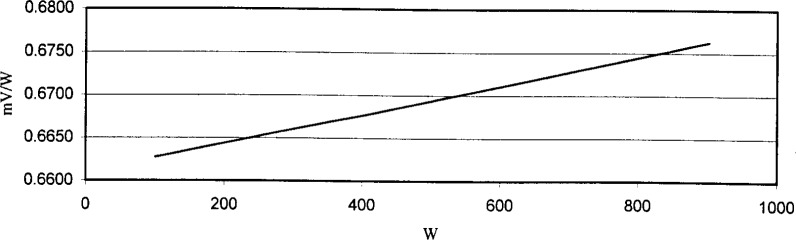
Positive nonlinear behavior of the detector.

Figures 2 and 3 plot the results from detector nonlinearity measurements performed on two detectors. The horizontal axis shows incident optical power levels and the vertical axis shows the detector’s responsivity in mV/W. The detector in Fig. 2 shows negative nonlinear behavior, or saturation in which the detector’s responsivity decreases as the incident optical power increases. Figure 3 shows positive nonlinear behavior, or supralinearity, in which the responsivity increases as the incident power increases. If each detector had been perfectly linear, the responsivity at each power level would be the same and, consequently, the data points would all lie on a horizontal line in each graph.

## 5. Measurement Uncertainty

To establish uncertainty limits of detector nonlinearity measurements, uncertainty components are grouped into two categories: Type A, whose magnitudes are obtained statistically from a series of measurements, and Type B, whose magnitudes are determined by scientific judgment. [8]. There are two major components of Type B uncertainty in detector nonlinearity measurements. The first is the laser/system instability (about 0.5 %). Variation of the laser power, or of the beam’s intensity profile can create several instability effects in the detector nonlinearity measurement system. For example, a rapid power shift may cause a monitoring error due to unequal time constants of the monitor detector and test meter, and changes in the high order transverse mode content of the laser beam may also generate monitoring error due to spatial non-uniformity of the monitor detector. The second Type B component is the polynomial truncation (about 0.1 %) performed in the polynomial curve fitting process.

There are two major components of uncertainty arising from Type A effects in the detector nonlinearity measurements. The first, stems from the chopper wheel ratio measurements, which are normally performed at three different radii along the open sector and, have a typical standard deviation under 0.05 %. The second Type A component is the test meter measurement repeatability, which includes noise in the data from both the test detector and the monitor detector. In addition the expanded uncertainty (*k* = 2) [9] of a typical NIST high-power laser calibration is about 1 %, so that the overall expanded uncertainty (*k* = 2) of a typical NIST high-power laser calibration with the non-linearity measurement is about 1.3 %.

## 6. Conclusions

NIST has extended the power range capacity of its high-power laser measurement program by developing a conceptually simple, nonlinearity measurement system for detector characterization. In conjunction with the NIST absolute standard calorimeters, this new system allows NIST to perform accurate power meter calibration over a much larger power range than previously existed at our facility. The system has been successfully demonstrated and used for power meter calibrations for industrial customers. This technique was recently used to perform an off-site detector calibration for a U.S. industrial laser manufacturer whose CO_2_ laser source was used with the NIST non-linearity system described above to characterize the nonlinearity of their detector over the power range of 400 W to 6000 W.
